# Development of the Tobacco Tactics logo: From thumb prints to press

**DOI:** 10.1186/1617-9625-10-6

**Published:** 2012-04-19

**Authors:** Lee A Ewing, Carrie A Karvonen-Gutierrez, Devon Noonan, Sonia A Duffy

**Affiliations:** 1 Ann Arbor VA Center for Clinical Management Research, Health Services Research and Development, 2215 Fuller Rd, Ann Arbor, MI 48105, USA; 2 University of Michigan, School of Nursing, 400 North Ingalls Building, Ann Arbor, MI 48109, USA; 3 Ann Arbor VA Center for Clinical Management Research, Health Services Research and Development, and the University of Michigan, School of Nursing, Departments of Otolaryngology and Psychiatry, 2215 Fuller Rd, Ann Arbor, MI 48105, USA

**Keywords:** Marketing, Advertising, Tobacco, Smoking

## Abstract

**Background:**

The purpose of this study was to describe the development and evaluation of the image-based Veterans Affairs (VA) Tobacco Tactics program logo and campaign character using principles of social marketing.

**Methods:**

Four cross-sectional surveys with open- and closed-ended questions were used to gather participant demographic information, smoking behavior, and feedback on the development and evaluation of the Tobacco Tactics program logo and campaign character. The first 3 surveys were conducted with 229 veterans, visitors, and staff to obtain feedback for the final logo and character choice. The fourth survey was conducted with 47 inpatient veteran smokers to evaluate the Tobacco Tactics manual which was illustrated with the logo and campaign character. Descriptive statistics and bivariate analyses comparing demographic characteristics and tobacco use variables to opinions about the pictures for each round of testing were computed.

**Results:**

After three rounds of testing to modify the logo and character choices based on participant feedback and survey data, the bulldog logo was chosen to represent the VA Tobacco Tactics program as it was viewed as strong and tough by the majority of participants. About 80% of the participants rated the manual highly on items such as logo, color, and pictures/illustrations. Almost 90% said they would recommend the manual to someone trying to quit smoking.

**Conclusion:**

Social marketing techniques that include consumer feedback to develop appealing tobacco cessation campaigns can increase consumer engagement and enhance the development of compelling tobacco cessation campaigns to compete with the influential marketing of tobacco companies.

## Background

Although smoking rates among veterans have decreased from 33% to 22.2% in recent years [1,2], smoking remains a problem in the Department of Veterans Affairs (VA). While the VA has done a great deal to address smoking among veterans including outpatient groups, electronic medical record reminders, and easy access to cessation medications, little has been done to develop a unified strategy to address inpatient smoking across VA hospitals. In 2006 in Veterans Integrated Service Network 11, the Tobacco Tactics program was implemented and evaluated at two large Midwestern VA hospitals using a third hospital as a control [3]. Tobacco Tactics is a nurse-based inpatient smoking cessation program that includes brief physician advice, bedside nurse counseling, a patient smoking cessation video and workbook, pharmaceutical management, and follow-up telephone calls by hospital volunteers. Prior to the rollout of the Tobacco Tactics program, social marketing techniques were used to develop a veteran-centric logo and campaign character to be used on all of the cessation materials so that the program would have brand identity and be appealing to veterans.

Social marketing is the planning and implementation of programs designed to bring about social change using concepts from commercial marketing including the "4 Ps": 1) Create an enticing "Product" (i.e. the package of benefits associated with the desired action); 2) Minimize the "Price" the t.arget audience believes it must pay in the exchange; 3) Make the exchange available in "Places" that reach the audience and fit its lifestyles; and 4) “Promote” the exchange opportunity with creativity and through channels and tactics that maximize desired responses [4]. A central component of social marketing is listening to the needs and desires of the target audience and building the program based on their feedback. It is important to evaluate the competition or rival offerings when developing a program.

Many public health campaigns and interventions have used advertising based on social marketing techniques to publicize and promote their programs [5-7]. Using social marketing techniques, these programs develop campaign names, logos, and messages based on focus groups, interviews, and known effective marketing strategies to engage their target population. For example, mass media campaigns have been developed by many states as part of a comprehensive tobacco control program. These campaigns more than likely have contributed to reducing smoking rates [8] by raising awareness and educating the public about the harmful effects of smoking on a population-wide level.

Anti-tobacco organizations have the challenge of developing campaigns that can compete with the multi-billion dollar tobacco industry. In 2006, the major tobacco companies spent $12.4 billion on advertising and promotions in the United States [9]. Tobacco advertisements use appealing imagery to promote smoking as a desirable behavior among target populations. For example, Marlboro ads appeal to males and promote masculinity; Virginia Slims ads are designed to appeal to women who prefer to be slender and feel independent and empowered; and Newport ads that depict babbling brooks in the background are designed to appeal to African Americans who value freshness [10,11]. Animal campaigns have been extremely successful in attracting consumer attention like the Joe Camel campaign which portrayed the camel as a “smooth character” that appealed to a younger audience [12]. More recently, innovative, new advertisements for Camel No. 9 cigarettes have been targeted at young women promoting the cigarettes as “light and luscious” [13].

To compete with the enticing messages produced by tobacco advertisers and counter the harmful effects of tobacco messaging, novel and strategic smoking cessation campaign strategies are needed. This is especially true for veterans who have a history of receiving cigarettes as part of their rations. Using principles of social marketing, the purpose of this study was to describe the development and evaluation of the image-based VA Tobacco Tactics program logo and campaign character that was rolled out in two Midwestern VAs.

## Methods

### Design

Using social marketing techniques, four cross-sectional surveys were conducted. The first 3 surveys were for formative evaluation of the Tobacco Tactics logo and campaign character. Formative evaluation is done with a small group of people to "test run" various aspects of instructional materials. The fourth survey was a final summative evaluation of the Tobacco Tactics manual which was illustrated with the logo and campaign character. Summative evaluation is done to determine if the instructional materials do what they are designed to do [14]. Institutional Review Board approval was received from the Ann Arbor and Detroit VAs.

### Setting and sample

Participants were recruited from the Ann Arbor and Detroit VA hospitals (except for round 2 which, due to logistic constraints was conducted in Ann Arbor only). Included in the first three rounds were veteran patients, visitors, and staff that were willing to complete the anonymous survey (N = 229). Round four included 47 inpatient veteran smokers that used the Tobacco Tactics manual to quit smoking.

### Procedures

A graphic design firm, Allen Wayne Ltd., was hired to illustrate the logo and character for the VA Tobacco Tactics program. Initially, the firm developed thumb sketches of 4 logos and 10 variations of a character that would be portrayed doing different activities related to quitting smoking for use in the Tobacco Tactics manual for patients. Formative evaluation for the first 3 rounds was conducted by distributing surveys to veteran patients, visitors, and staff around November 15, 2007, which was the date of the American Cancer Society’s Great American Smokeout. Research staff was stationed at strategic points around the hospital, such as the cafeteria and smoking shelter. Nurse managers and research nurses who were familiar with the Tobacco Tactics program also distributed surveys to staff on each unit. Each successive round of surveys used feedback from the previous round to narrow down the choices for the final logo and campaign character. Summative evaluation was conducted by placing an evaluation survey, $3 canteen coupon, and postage-paid return envelope in the Tobacco Tactics manual given to inpatient veteran smokers.

### Measures

For all four rounds of surveys, both open- and closed-ended questions were used to evaluate the Tobacco Tactics logo, character, and cigarette logos. Open-ended questions included: 1) What three words best describe this picture?; 2) What things about this picture would convince people to stop smoking?; and 3) How would you change the picture to better convince people to stop smoking? Closed-ended questions included: 1) Which logo and character best represents the Tobacco Tactics program? and 2) Which logo and character best conveys the message to quit smoking? To evaluate the competition, participants were also asked about the logos of three major cigarette brands (i.e. Marlboro, Camel, and Newport) and which logo most conveys smoking. Open-ended questions on the summative evaluation survey included: 1) What do you think about the pictures that are shown throughout the manual?; 2) Please comment on any aspects of the manual that you found to be appealing; and 3) Please provide any thoughts you have about how this manual could be improved. Closed-ended questions included: 1) How would you rate the Tobacco Tactics manual?; 2) The manual was easy to read and understand (strongly disagree to strongly agree); 3) The manual was enjoyable to read (strongly disagree to strongly agree); 4) How helpful did you find each of the following sections of the manual? (i.e. smoking medications, behavioral management, and paper-and-pencil exercises); and 5) Would you recommend this manual to someone else who is thinking of quitting smoking? Participants were also asked to rate the manual on its cover design, logo, pictures/illustrations, and color. Demographic information (i.e. age, sex, race, education, marital status, and position – staff vs. patient) and tobacco use status questions were collected for all rounds.

### Data analysis

Descriptive statistics were calculated for the variables. Bivariate analyses including Chi-square and Fisher’s Exact tests were used to examine relationships among the demographic and tobacco use variables and opinions about the pictures. All statistical tests were considered significant at a level of p < 0.05. All analytic statistics were run using SAS V9.2 statistical software. Content analysis was conducted with the data from the open-ended questionnaires by two independent reviewers to determine the presence of certain words or concepts within the text. This data was then corroborated to determine the final themes or concepts from the analysis.

## Results

### Round one

**Figure 1:**
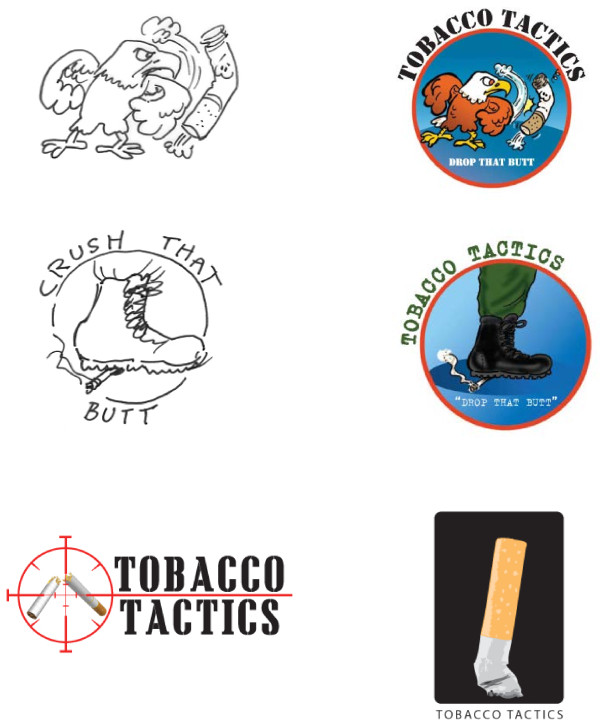
First round logo options.

Demographic information for the first three rounds is shown in Table1. As shown in Table 2, over 37% of those participants chose the target logo as their first choice over a boot stomping a cigarette, an eagle punching a cigarette, and a cigarette butt. See Figure 1 for the four logo options. The most common words to describe the target logo were “stop/quit smoking,” followed by “target/taking aim,” “breaking the habit,” and “kill/dead.” Staff were more likely than patients to think the target logo gave a violent message (p < 0.05) which may be problematic for veterans with post-traumatic stress disorder. About 27% preferred the eagle logo because it represented America, but others thought it looked aggressive and did not want us to “mess with” this American symbol. Participants described the eagle as “aggressive,” “strong,” and “tough.” Males were more likely to favor the eagle logo while females were more likely to favor the cigarette butt logo and the boot logo (p < 0.05). Participants thought that the image of a cigarette breaking would convince someone to quit smoking. There were no other significant differences between demographic and smoking variables and the choice of logo or campaign character. In this round, participants were asked what words best describe the three major cigarette brand logos (i.e. Marlboro, Camel, and Newport). The most commonly cited words were “royalty” (Marlboro), “Middle Eastern/Exotic” (Camel), and “color” (Newport).

**Table 1:** Demographic information among respondents in rounds 1, 2, and 3

	Round 1* (N = 72)		Round 2** (N = 62)		Round 3* (N = 95)	
	**N**	**%**	**N**	**%**	**N**	**%**
**Smoking status**						
Currently smoke	31	44.93	16	26.23	32	33.68
Quit in last year	3	4.35	6	9.84	12	12.64
Quit over 1 year ago	14	20.29	15	24.59	19	20.00
Never smoked	21	30.43	24	39.34	32	33.68
**Age**						
Less than 35 years	9	13.24	7	12.28	20	22.99
35-44 years	13	19.12	8	14.04	14	16.09
45-54 years	27	39.71	27	47.37	18	20.69
Greater than 55 years	19	27.94	15	26.32	35	40.23
**Sex**						
Male	37	54.41	31	53.45	54	58.06
Female	31	45.59	27	46.55	39	41.94
**Race/ethnicity**						
White	38	56.72	50	81.97	52	57.14
Non-White	29	43.28	11	18.03	39	42.86
**Education**						
High school or less	12	17.15	9	15.00	21	22.58
Some college	34	48.57	28	46.67	45	48.39
4-year college or more	24	34.29	23	38.33	27	29.03
**Marital status**						
Married	29	42.03	25	41.67	46	48.94
Separated/Widowed/ Divorced	24	34.79	22	36.66	28	29.78
Never Married	16	23.19	13	21.67	20	21.28
**Patient or staff**						
Patient	26	36.62	24	39.34	32	35.16
Staff	39	54.93	36	59.02	54	59.34
Visitor	2	2.82	----	----	5	5.49
Other	4	5.63	----	----	----	----
Both	----	----	1	1.64	----	----
**Staff position**						
LPN	3	7.89	3	8.33	4	7.84
RN	22	57.89	21	58.33	18	35.29
Other	13	34.21	12	33.34	29	56.86

*Rounds 1 and 3 included participants from Ann Arbor and Detroit.

**Round 2 included participants from only Ann Arbor.

**Table 2:** Logo preferences among respondents in rounds 1, 2, and 3

	Round 1* (N = 72)		Round 2** (N = 62)		Round 3* (N = 95)	
	**N**	**%**	**N**	**%**	**N**	**%**
**Favorite logo**						
Eagle	17	26.56	----	----	----	----
Cigarette butt	12	18.75	----	----	----	----
Target	24	37.50	----	----	----	----
Army boot	11	17.19	----	----	----	----
No selection	8	----	----	----	----	----
**Which of the pictures best conveys message of quitting smoking?**						
Bulls-eye logo	----	----	18	31.58	----	----
No graphic/Words only logo	----	----	9	15.79	----	----
Viewfinder logo	----	----	30	52.63	----	----
**Which of the 4 animals would convince people to quit smoking?**						
Bear	----	----	17	29.31	----	----
Bulldog	----	----	21	36.21	----	----
Gorilla	----	----	15	25.86	----	----
Lion	----	----	5	8.62	----	----
**Which of the 4 human figures would convince people to quit smoking?**						
Human 1	----	----	7	12.50	----	----
Human 2	----	----	5	8.93	----	----
Human 3	----	----	5	8.93	----	----
Human 4	----	----	39	69.64	----	----
**Which logo most conveys smoking?**						
Marlboro logo	----	----	20	32.26	29	31.52
Camel logo	----	----	42	67.74	56	60.87
Newport blue swoosh logo	----	----	0	0.00	7	7.61
**Which of the 2 characters would best represent the VA Tobacco Tactics program?**						
Bulldog	----	----	----	----	32	35.56
Drill sergeant	----	----	----	----	58	64.44

*Rounds 1 and 3 included participants from Ann Arbor and Detroit.

**Round 2 included participants from only Ann Arbor.

### Round two

**Figure 2:**
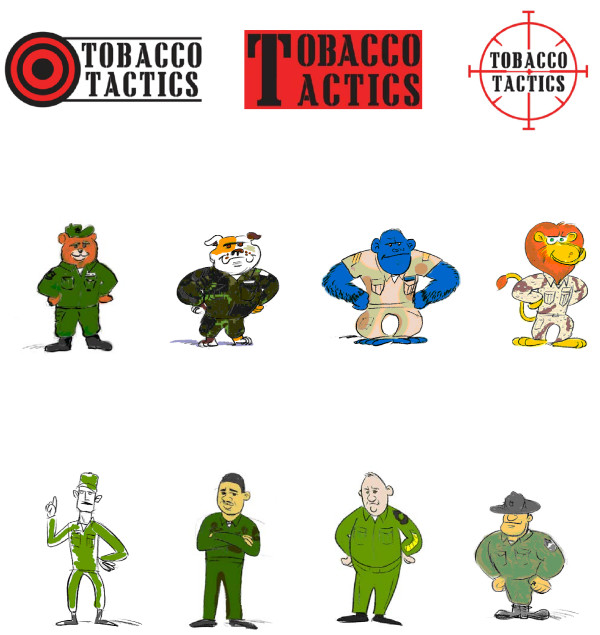
Second round logo and character options.

Based upon the survey results in the first round, two versions of the target logo were designed to be less violent and the picture of the cigarette was removed from the target as it may actually trigger smoking (see Figure2). As shown in Table2, approximately 53% preferred the viewfinder target logo compared to the bulls-eye and words-only logos. Content that emerged from the analysis were that many veterans and staff thought that the viewfinder target logo indicated an action to quit smoking, e.g. “set your sights on quitting” and “taking aim at the problem.” Others commented on the color scheme of the logos, including that the color red was convincing in the logos. Staff continued to be more likely than patients to believe the viewfinder and bulls-eye logos were “violent” or sent “unclear messages.” Whites were more likely to favor the bulls-eye logo compared to non-Whites (p < 0.05). In this round, participants were asked which major cigarette brand logo most conveys smoking; about two-thirds of the participants thought the Camel logo was most likely to convey smoking compared to the Marlboro and Newport logos.

In addition, in this round of testing, four animal and four human military-type cartoon characters were illustrated and introduced to the participants for feedback (see Figure2). Approximately 36% preferred the bulldog compared to the bear (29%), gorilla (26%) and lion (9%). The bulldog was viewed as “strong,” “tough,” and “confident.” Nearly 70% preferred the drill sergeant compared to the other 3 military-type characters; words used to describe the drill sergeant included “authority,” “strong,” “tough,” “healthy,” and “serious.” There were no significant differences between demographic and smoking variables and the choice of logo or campaign character.

### Round three

In the final round of surveys, participants were asked to choose between the two target logos, drill sergeant and bulldog characters, and the two Tobacco Tactics logos with characters. As shown in Table2, the drill sergeant and bulldog were both viewed as strong and tough and 64% preferred the drill sergeant and 36% preferred the bulldog. There were no significant differences between demographic and smoking variables and the choice of logo or campaign character. Almost two-thirds of the participants again chose the Camel logo as the most likely to convey smoking compared to the Marlboro and Newport logos.

Qualitative analyses revealed that participants described the bulldog as “strong,” “tough,” “determined,” “cute,” and “American/patriotic.” Many participants liked the “toughness” and “color” of the drill sergeant. His “smile” and perception of healthiness conveyed the message to quit smoking. Unfortunately, several veteran participants stated that, while in the service, their drill sergeant told them to “smoke 'em if you got 'em.”

### Round four

**Figure 3:**
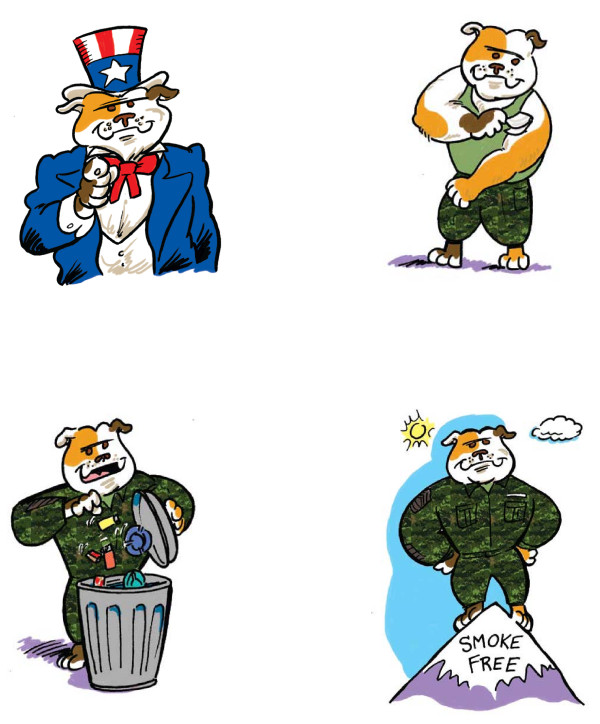
Example Illustrations from the Tobacco Tactics manual.

Table3 shows the summative evaluation of the illustrated manual used by 47 inpatient veteran smokers. About 80% of the participants rated the manual highly on all items. Almost 90% said they would recommend the manual to someone trying to quit smoking. Positive comments included “stands out and grabs your attention,” “bright, reminds me of good old Uncle Sam,” “the book is illustrated well, people relate to humorous help while they’re reading,” “excellent cartooning - both humorous and commanding (first sergeant dog),” “cute and cuddly puppy,” and “pictures were easy for me to understand and very helpful.” One participant said the “bulldog is a Marine Corps thing; but as an Air Force guy I can take it!” while another said “Army - don't like marine dog!” Other comments included: “gear more towards females, add a poodle with a pink ribbon” and “they were a little bit for kids.” See Figure3 for example illustrations from the Tobacco Tactics manual.

**Table 3:** Evaluation of the Tobacco Tactics logo and manual from Ann Arbor (N = 29) and Detroit (N = 18)

	**N**	**Percent**
**In general, the Tobacco Tactics manual is**		
Very Poor	1	2.27
Poor	1	2.27
Neutral	4	9.09
Good	23	52.27
Excellent	15	34.09
**Cover design**		
Very Poor	0	0
Poor	3	6.82
Neutral	6	13.64
Good	19	43.18
Excellent	16	36.36
**Logo**		
Very Poor	0	0
Poor	1	2.33
Neutral	8	18.60
Good	20	46.51
Excellent	14	32.56
**Pictures and illustrations**		
Very Poor	0	0
Poor	0	0
Neutral	7	15.91
Good	24	54.55
Excellent	13	29.55
**Color**		
Very Poor	0	0
Poor	0	0
Neutral	6	13.95
Good	18	41.86
Excellent	19	44.19
**Manual was easy to read**		
Strongly Disagree	0	0
Disagree	1	2.33
Neutral	2	4.65
Agree	20	46.51
Strongly Agree	20	46.51
**Manual was enjoyable to read**		
Strongly Disagree	1	2.44
Disagree	0	0
Neutral	9	21.95
Agree	18	43.90
Strongly Agree	13	31.71
**How helpful was section on Smoking Medications?**		
Extremely Unhelpful	0	0
Somewhat Unhelpful	1	2.17
Neutral/Undecided	5	10.87
Somewhat Helpful	22	47.83
Extremely Helpful	18	39.13
**How helpful was section on Behavioral Management?**		
Extremely Unhelpful	0	0
Somewhat Unhelpful	2	4.35
Neutral/Undecided	10	21.74
Somewhat Helpful	17	36.96
Extremely Helpful	17	36.96
**How helpful were the Paper-and-Pencil exercises?**		
Extremely Unhelpful	0	0
Somewhat Unhelpful	4	8.70
Neutral/Undecided	9	19.57
Somewhat Helpful	21	45.65
Extremely Helpful	12	26.09
**Recommend to someone else who is thinking of quitting smoking**		
Strongly Disagree	1	2.17
Disagree	2	4.35
Neutral	3	6.52
Agree	10	21.74
Strongly Agree	30	65.22
**Smoking status**		
Current Smoker	31	67.39
Former Smoker	11	23.91
Never Smoker	4	8.70
**Age**		
Less than 44 Years	5	10.87
45-54 Years	18	39.13
55-64 Years	17	36.96
Greater than 64 Years	6	13.04
**Sex**		
Male	42	91.30
Female	4	8.70
**Race**		
White	26	56.52
Non-White	20	43.47
**Education**		
High school or less	17	36.96
Some college	23	50.00
4-year college or more	6	13.04

## Discussion

**Figure 4:**
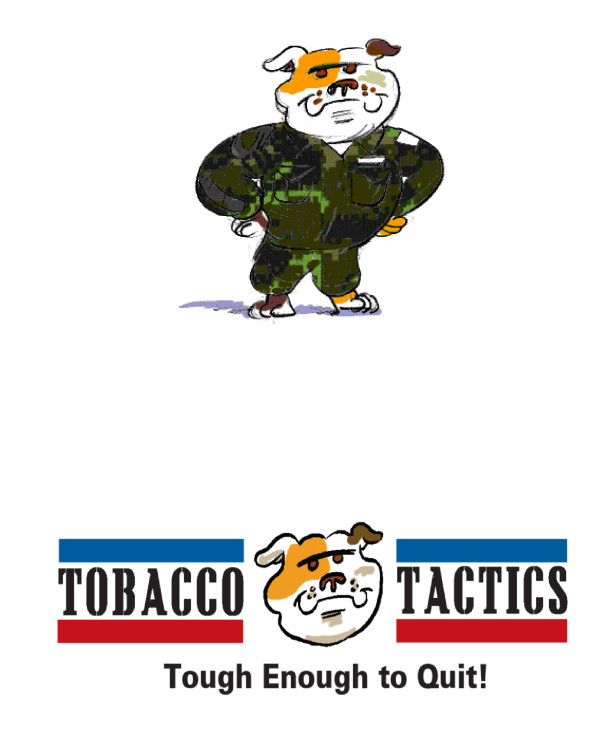
Final logo and character.

Faced with the dilemma of conflicting quantitative data and qualitative data, the team deliberated and chose the bulldog, viewed as tough and strong, as the final logo and character for the Tobacco Tactics program (see Figure4) for the following reasons. First, it was risky to choose a logo and character such as the drill sergeant that some veterans associated with smoking. Second, there is much anecdotal and some empirical evidence that pets, particularly dogs, are important to veterans [15,16]. Third, since animal campaigns (e.g. Coca-Cola Bears) have been shown to be highly successful in marketing products and the animal-based Camel logo was noted by participants as most likely to convey smoking, the animal-based bulldog logo was chosen as a counter-advertising strategy.

A picture logo was chosen rather than a words-only logo as research has shown that people remember visual images much better than words because pictures are more attention-getting and easier to process [17]. The presence of pictures in advertisements has the ability to influence consumers’ cognitive activity while viewing the advertisement [17] and enhance memory of the brand name or target information [5]. Pictures often provoke thought and help connect the material to peoples’ own experiences [18]. In addition to pictures, campaigns that use bright colors, humor, and cartoon characters help people recognize familiar brand names, logos, and characters and associate them with certain messages [5]. While a few participants did not like the illustrations, the vast majority thought the bulldog is visually appealing, recognizable, humorous, and easy to remember.

Humor is a popular persuasion device for anti-smoking campaigns, and generates more favorable responses towards a particular message and facilitates persuasion by attracting receiver attention [19]. Little has been written about the effects of humor in anti-smoking campaigns for veterans. However, humor has been found to assist veterans in releasing tension brought on by their often times traumatic experiences in combat and can be used to help cope with emotionally stressful events [20]. Humor helps veterans humanize the healing process, manage stress, and engage in creative problem-solving skills [20]. This may be particularly helpful for veterans who suffer from post-traumatic stress disorder or depression that are trying to quit smoking.

This logo pairs a memorable bulldog character with a catchy slogan (“Tobacco Tactics: Tough Enough to Quit!”), both of which facilitate the connection between the Tobacco Tactics program and quitting smoking. Linking smoking cessation to a visual symbol and slogan helps to create a brand identity that veterans can easily associate with quitting smoking. Compelling images, such as the bulldog, reinforce other components of the message and look more appealing than competing risky health behaviors [21].

Tobacco companies have done extensive research to ensure that their brand stays on consumer’s minds and facilitates use of their product [22]. Public health initiatives geared towards tobacco control must do the same to be appealing to the target population [23]. Similar to other public health campaigns that have designed simple, recognizable logos for print materials to increase public demand for the program [24,25], the Tobacco Tactics bulldog logo and character is now used on cessation materials distributed around the hospital.

Involving veterans in branding the Tobacco Tactics program is a strategic move to invest veterans in a product that they can identify with and use to aid in tobacco cessation. The greater the personal identification towards a product, the greater the motivation to recommend it [26], which could explain why the vast majority of veterans agreed that they would recommend the manual to others. Visitors and staff were also asked their opinions and, in this case, staff had strong feelings against the target logo as they felt it depicted violence. Since staff would be the ones using the materials to work with patients on smoking cessation, it was important that they also found the logo and character appealing. This makes the case for involving not only the target population, but all stakeholders in these decisions.

### Limitations

Participants were recruited from Midwest VA hospitals which may limit the generalizability of the results to veterans in other geographic areas. Small convenience samples were used to evaluate the logo and character which may have biased the results. Nonetheless, these techniques, used on a small scale in this study, are similar to those used by advertisers to develop campaigns.

## Conclusions

Social marketing techniques that include consumer feedback to develop appealing tobacco cessation campaigns can increase consumer engagement and enhance the development of compelling tobacco cessation campaigns to compete with the influential marketing of tobacco companies. The Tobacco Tactics manual has been re-illustrated to be non-veteran-centric and is currently being used in a National Institutes of Health study evaluating the dissemination of inpatient smoking cessation interventions in the general population. Evaluating the manual in a non-VA population will increase generalizability and provide further information about what smokers find helpful to aid them in tobacco cessation.

## Competing interests

The authors declare that they have no competing interests.

## Authors’ contributions

SD conceived the idea for the study. LE participated in data collection and qualitative data analysis. LE and SD drafted the initial manuscript. CKG performed the statistical analyses. DN reviewed and edited the manuscript. All authors read and approved the final manuscript.
